# Electrospun Hydrophobic Interaction Chromatography (HIC) Membranes for Protein Purification

**DOI:** 10.3390/membranes12070714

**Published:** 2022-07-18

**Authors:** Shu-Ting Chen, Sumith Ranil Wickramasinghe, Xianghong Qian

**Affiliations:** 1 Department of Chemical Engineering, University of Arkansas, Fayetteville, AR 72701, USA; sc086@uark.edu (S.-T.C.); swickram@uark.edu (S.R.W.); 2 Department of Biomedical Engineering, University of Arkansas, Fayetteville, AR 72701, USA

**Keywords:** electrospun membranes, protein purification, hydrophobic interaction chromatography

## Abstract

Responsive membranes for hydrophobic interaction chromatography have been fabricated by functionalizing poly(N-vinylcaprolactam) (PVCL) ligands on the substrate of electrospun regenerated cellulose nanofibers. Both static and dynamic binding capacities and product recovery were investigated using bovine serum albumin (BSA) and Immunoglobulin G (IgG) as model proteins. The effects of ligand chain length and chain density on static binding capacity were also studied. A static binding capacity of ~25 mg/mL of membrane volume (MV) can be achieved in optimal ligand grafting conditions. For dynamic binding studies, protein binding capacity increased with protein concentration from 0.1 to 1.0 g/L. Dynamic binding capacity increased from ~8 mg/mL MV at 0.1 g/L BSA to over 30 mg/mL at 1.0 g/L BSA. However, BSA recovery decreased as protein concentration increased from ~98% at 0.1 g/L BSA to 51% at 1 g/L BSA loading concentration. There is a clear trade-off between binding capacity and recovery rate. The electrospun substrate with thicker fibers and more open pore structures is superior to thinner fibrous membrane substrates.

## 1. Introduction

The rapid development of biopharmaceutical products has led to an increased demand for purification of high product titer feed, and regulatory agencies have implemented strict requirements for product purity [1]. However, downstream purification accounts for 50–80% of the entire production cost [2]. There is a severe bottleneck for cost-effective purification of biopharmaceuticals due to the dramatic increase in the number of products and the increased product titer and purity requirements [3]. Significant efforts have been made in recent years to overcome these challenges in order to improve the efficacy of purification during downstream processing.

Hydrophobic interaction chromatography (HIC) is often used as a chromatographic polishing step to remove the remaining host cell proteins (HCP), product aggregates and other more hydrophobic impurities during the downstream purification of biologics such as monoclonal antibodies (mAbs), Fc-fusion proteins and other recombinant therapeutic proteins as well as many other biologics including hormones, vaccines, growth factors and interferons [4,5]. The mechanism for HIC purification comes from the different hydrophobic binding interaction strengths between molecules with different hydrophobicity in the feed and the hydrophobic ligand immobilized on the stationary phase [4]. As hydrophobic interaction is modulated by the ionic strength of the solution, high ionic strength buffer is often used to bind the hydrophobic species in the feed whereas low ionic strength buffer is often used to elute the bound species. Currently, HIC is often operated in the flow-through mode where the product of interest passes through the stationary phase and impurities, including aggregates and other more hydrophobic species, are captured by the immobilized ligands. In addition, our previous studies [2,6,7,8] show that salt type and salt concentration, as well as ligand chain length and chain density, affect overall chromatographic performance of HIC membranes. Both protein binding capacity and recovery are observed to depend on both mobile phase conditions and stationary phase properties as well as to be protein dependent [6,8,9,10].

Packed-bed column chromatography has been widely used in downstream purification of proteins, nuclei acids and other biologics [2]. However, one major drawback of packed-bed column chromatography is the slow pore-diffusion which severely restricts its separation efficiency. The diffusion of targeted products to the ligands on the chromatographic bed is a slow process leading to a dramatic drop in binding capacity as the feed flow rate increases. Besides diffusion limitation, packed-bed chromatography also suffers from large buffer consumption as well as extra costs for packing and testing.

An alternative approach is to use adsorptive membrane chromatography during downstream processing [2]. Adsorptive membranes, known as membrane adsorbers, are macroporous membranes functionalized by ligands attached on the membrane pore surface to remove containments, such as product aggregates, viruses and DNAs [11]. Compared to resin-based chromatography, the pore diffusion limitation is eliminated in membrane adsorbers where convection becomes the dominant transport mechanism [12]. Moreover, the operation can be performed at relatively low pressure, which reduces denaturation and aggregation of the sensitive biologics. Buffer usage of membranes is lower than resins due to reduced void volume.

Membranes have substantially lower material cost compared to packed beds. Single-use membrane processes greatly reduce the cost of revalidation. In addition, the membrane system is easier to scale up and the cost of packing and testing is subsequently reduced significantly. Traditionally, membrane-based purification is always limited by its low capacity. Recent advances in materials engineering have led to high-capacity membranes that can now compete with resins [13,14]. Currently, there is interest in developing HIC membrane chromatography in the bind-and-elute mode for protein fractionation [5,7,9,12,14,15]. However, application of HIC has been limited due to the overall low capacity of the ligands and the efficiency of eluting the bound proteins [15].

Thermo-responsive polymer, such as poly(N-isopropylacrylamide) (PNIPAM) and poly(N-vinylcaprolactam) (PVCL), has a low critical solution temperature (LCST), above which the polymer adopts a collapsed hydrophobic conformation and below which the polymer has a coil-like hydrophilic conformation [16,17]. The LCST of the polymer is also affected by the salt type and salt concentration in the solution [16,18,19,20,21,22]. The reduction in LCST follows the Hofmeister series and sometimes an inverse Hofmeister series [5,18,21]. The microscopic mechanisms of the Hofmeister effect are not completely understood yet; however, the impact of salt ions and salt concentrations on the properties of polymers and biological molecules is tremendously significant. PVCL has a LCST between 30 °C and 50 °C in water [16,23]. Transition temperature is also affected by its molecular weight and polymer concentration in aqueous solution due to the presence of the bulky seven-member ring on the polymer side chain. The polymer switching to a hydrophobic state will promote stronger hydrophobic interaction and thus binding of the protein. In contrast, the polymer transitioning to a hydrophilic state will promote protein desorption [18,24]. Both PVCL and PNIPAM have been investigated for application as HIC ligands for protein purifications [4,5,6,7,8,10,24,25,26,27]. However, the advantages of using PVCL are its biocompatibility and low toxicity. The hydrolysis of PNIPAM at acidic or basic conditions could generate low molecular weight amines which are toxic to biological systems. On the other hand, PVCL does not produce small amine molecules since the amide bond is located on its seven-member ring [6,16].

During the past few years, we have investigated the responsive HIC membranes by grafting PVCL chains to the surface of regenerated cellulose (RC) membranes [6,8,10,23]. The static binding capacity for BSA at around 10–15 mg/mL MV (membrane volume) and dynamic binding capacity (DBC) at around 8–12 mg/mL MV in 1.8 M (NH_4_)_2_SO_4_ solution are already comparable, if not better, than the commercial HIC membranes available. The effects of PVCL chain length and chain density on protein binding and recovery are also investigated. Our previous results suggest that there is a trade-off between capacity and recovery for the PVCL ligands immobilized on the flat-sheet commercial RC membrane substrates. Longer and denser chains lead to higher binding capacity but reduced recovery, due largely to constriction of the pores during elution. In order to further improve the performance of these responsive HIC membranes, it seems that a more porous membrane substrate with a higher surface area or higher surface-to-volume ratio than that of commercial RC membranes is more desirable. Previously, PNIPAM and its thermo-responsive copolymers were investigated for antibody purifications [25,26,27]. The functionalized copolymers possess negatively-charged sulfonic acid groups at neutral pH. As a result, these polymers can selectively bind antibodies based on both charge and hydrophobic interactions.

Membranes made from electrospun fibers have high porosity with a high surface-to-volume ratio [28]. Electrospun membranes have demonstrated broad applications in water treatment, tissue engineering and protein purifications with ion-exchange membranes [14,29,30,31,32,33,34,35,36]. We previously fabricated weak electrospun anion-exchange membranes, for protein capture [14], as well as mixed matrix membranes, for ammonium removal [36]. Compared to flat sheet membrane substrates, significantly higher binding capacity was obtained for protein capture [14]. However, there are no previously reported studies on the fabrication of electrospun HIC membranes. In order to overcome the current limitations of HIC membranes and to achieve higher protein binding capacity and better product recovery, electrospun RC membrane substrates were fabricated and used for grafting PCVL ligands using controllable atom-transfer radical polymerization (ATRP) while varying polymer chain density and chain length. The electrospun RC membrane substrates were characterized using Fourier transform infrared spectroscopy (FTIR), X-ray photoelectron spectroscopy (XPS) and scanning electron microscopy (SEM). The performance of these responsive electrospun HIC membranes was investigated to determine both static and dynamic binding capacities of BSA under industrially applied (NH_4_)_2_SO_4_ salt solutions. Finally, the membranes were used in the bind-and-elute mode chromatography of an industrial IgG_4_. The main objective of this research is to investigate electrospun HIC membranes for bind-and-elute application with both enhanced protein binding capacity and improved product recovery to meet the high product titer demands of the biopharmaceutical industry and the high product purity requirements of regulatory agencies.

## 2. Materials and Methods

### 2.1. Materials

N,N,N,N,N-pentamethyl diethylenetriamine (PMDETA, 99%), 2-bromo-2-methylpropionyl bromide (BIB, 98%), 2-hydroxyethyl methacrylate (98%), N-vinylcaprolactam (98%), 4-(dimethylamino) pyridine (DMAP, >99%), copper (I) chloride (Cu(I)Cl, >99.99%), copper (II) chloride (CuCl_2_, >99.99%) and cellulose acetate (CA, Mn ~30 kD) were obtained from Sigma-Aldrich (St. Louis, MO, USA). Triethylamine (TEA, >99%) and N,N dimethylacetamide (DMAc, 99%) were sourced from Alfa Aesar (Ward Hill, MA, USA). Acetonitrile (>99.8%), methanol (99.8%), acetone (>99.5%) and ammonium sulfate (proteomics grade) were purchased from VWR (Radnor, PA, USA). Boric anhydride and BSA were acquired from Avantor Performance Materials (Center Valley, PA, USA). Purified human IgG_4_ monoclonal antibody was donated by industry. Deionized (DI) water was generated using the Thermo Fisher Scientific (Waltham, MA, USA) DI water system.

### 2.2. Fabrication of Electrospun Membrane Substrate

In a typical experiment, cellulose acetate (13.8 wt%) was dissolved in an acetone/DMAc (2:1, *w*/*w*) mixture. It was then stirred at room temperature to obtain a homogenous solution and used as the casting solution. The casting solution was subsequently filled into a syringe pump and electrospun onto a piece of aluminum foil using an Electrospinning Apparatus. The applied voltage was kept at 12.5 kV while collector–needle tip distance was maintained at 15–20 cm. The flow rate of the syringe pump was kept at 0.3 mL/h. A nanofiber mat was obtained after collecting the nanofibers for 8 h. The thus electrospun cellulose acetate (CA) membrane was placed in a fume hood for one day to let the residual solvent evaporate. After annealing, the cellulose acetate membrane was peeled from aluminum foil and hydrolyzed in aqueous 0.1 M NaOH solution overnight without stirring to remove the acetyl groups from the acetate. Thereafter, the regenerated cellulose (RC) membrane samples were stored in a DI water bath until further use.

### 2.3. Ligand Grafting

**Figure 1:**
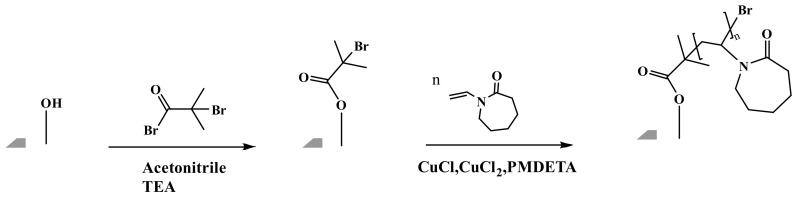
Reaction scheme for grafting poly(N-vinylcaprolactam) (PVCL) ligands on electrospun membranes with atom-transfer radical polymerization (ATRP).

The reaction scheme for grafting the PVCL ligand using ATRP is shown in Figure 1 and follows our previously published protocol [6]. Briefly, the electrospun RC membrane was dried in a vacuum oven for 8 h to remove residual moisture. The membrane was then immersed in the solution containing 40, 80 and 200 mM initiator BIB in acetonitrile for a predetermined period of time. In the meantime, a mixed solution was prepared by adding monomer VCL, catalyst CuCl and CuCl_2_ and conjugating ligand PMDETA into the 50:50 methanol/water *v*/*v*% solution. The ratio of VCL monomer, CuCl, CuCl_2_ and PMDETA was fixed at 200:1:0.2:2. Argon was used to degas the mixture solution for 15–20 min. The previously initiator-immobilized membrane was then placed into a clean round-bottom three-necked flask. Finally, the polymerization process was initiated by pouring the derived mixture solution into the flask under an inert gas environment. After a predetermined period of ATRP reaction, the modified membranes were rinsed three times using 50:50 *v*/*v*% methanol/water and then three times with DI water. The resultant membrane was kept in a DI water bath on the shaker to remove any residual solvent.

### 2.4. Characterization of the Functionalized HIC Membranes

All membrane samples were cleaned with DI water and dried in a vacuum oven (12.5 L, VWR International, Radnor, PA, USA) overnight prior to characterization. Static contact angle measurements were carried out at different ionic strengths of solution. Quintuplicate measurements were made to determine the standard deviation.

FTIR (IRAffinity, Shimadzu, MD, USA) was performed to determine characteristic functional groups of the modified membranes. XPS (Thermo Fisher Scientific Inc., Waltham, MA, USA) was used for analyzing the chemical compositions of the membranes before and after modification. SEM (FESEM S-4800, Hitachi Co., Tokyo, Japan) was used to characterize the surface fiber structures of both modified and unmodified membranes.

Grafting degree (GD) was calculated to quantitatively determine the amount of PVCL grafted. The membranes before and after modification were dried in a vacuum oven at 40 °C and the weight of the samples was thereafter measured. GD was calculated based on the following equation:

(1) $$\mathrm{GD}\;\left(\%\right)=\frac{W_{\mathrm{modified}}-W_{\mathrm{unmodified}}}{W_{\mathrm{unmodified}}}\times 100\%$$

### 2.5. The Static and Dynamic Binding Capacities of HIC Membranes

The fabricated HIC membranes were cut into 4.9 cm^2^ disks for the static binding capacity tests. Each membrane was placed in a 60 mL glass bottle from VWR (Radnor, PA, USA) and equilibrated for 1 h with the adsorption buffer (20 mM phosphate buffer with 1.8 M (NH_4_)_2_SO_4_ at pH 7.0, buffer A). Subsequently, the equilibrated membranes were challenged with model protein BSA at 5 different concentrations for 5 h at room temperature under gentle shaking. The final equilibrated BSA solutions were measured by UV absorbance at 280 nm with a UVVIS spectrophotometer (Thermo Scientific™ GENESYS 10S UV-Vis, Waltham, MA, USA) and their concentrations determined using a standard curve.

ÄKTA Pure (GE Healthcare Bio-Sciences Corp., Boston, MA, USA) was used to conduct the fast protein liquid chromatography (FPLC) experiments for dynamic binding capacity measurements. Unicorn software version 7.3 was used to automate experiments on BSA binding and elution. BSA solutions were prepared by dissolving 10 mg of BSA into 10 mL of buffer A, which contained 1.8 M (NH_4_)_2_SO_4_. IgG_4_ feed solutions were prepared by dissolving 1 mg of IgG_4_ into 10 mL of buffer A. Prior to the binding tests, both protein and buffer solutions were filtered using Whatman 0.2-μm Polyethersulfone (PES) membrane filters. Subsequently, four fabricated HIC membranes (0.04 mL membrane volume) were loaded into a stainless-steel flow cell (Mustang Coin module, Pall Corporation, Port Washington, NY, USA) equipped with two flow distributors to establish a uniform flow across all membranes. The membranes were equilibrated in the forward flow configuration in buffer A (adsorption buffer) for 10 min at 1 mL/min. A protein solution (1 mg/mL) was loaded onto the membrane for 10 min at a flow rate of 1 mL/min. Unbound proteins were then washed from the membrane surface using adsorption buffer (buffer A) for 5 min at 1 mL/min, followed by a step change to running the elution buffer (20 mM phosphate buffer with 0 mM (NH_4_)_2_SO_4_ at pH 7.0, buffer B) through the membranes at the same flow rate. The chromatographic run was stopped when the UV absorbance at 280 nm became constant. The elution and washing fractions, as well as the loading fraction, were collected and their protein concentrations were subsequently determined.

Both static and dynamic binding capacities were calculated based on the following equation:

(2) $$\mathrm{Binding}\;\mathrm{capacity}=\frac{\mathrm{Mass}\;\left(\mathrm{bound}\;\mathrm{protein},\;\mathrm{mg}\right)}{\;\mathrm{Membrane}\;\mathrm{Volume}\;\left(\mathrm{mL}\right)}$$

Protein recovery from the FPLC chromatographic run was determined using the equation below:

(3) $$\mathrm{Recovery}=\frac{\mathrm{Mass}\;\left(\mathrm{eluted}\;\mathrm{protein}\right)}{\;\mathrm{Mass}\;\left(\mathrm{bound}\;\mathrm{protein}\right)}\;\times 100\%$$

## 3. Results and Discussion

### 3.1. Physicochemical Properties of Fabricated HIC Membranes

**Figure 2:**
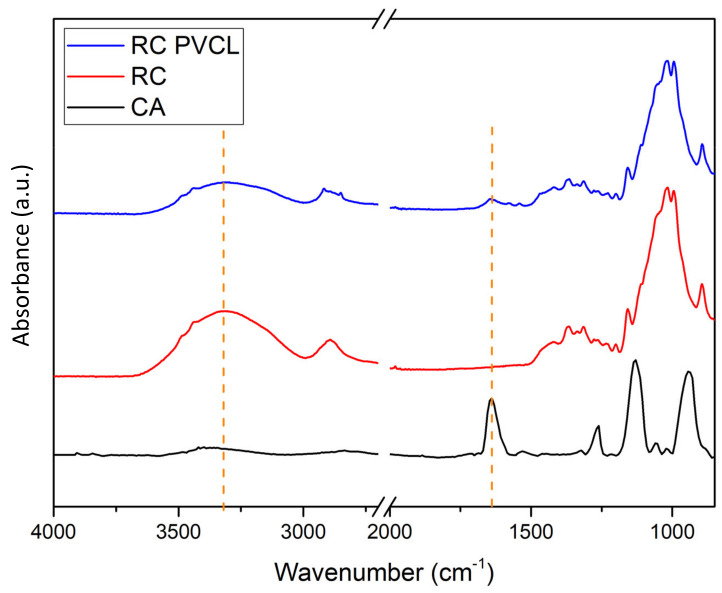
FTIR spectra of the cellulose acetate (CA), regenerated cellulose (RC) and surface–modified RC PVCL membranes.

**Figure 3:**
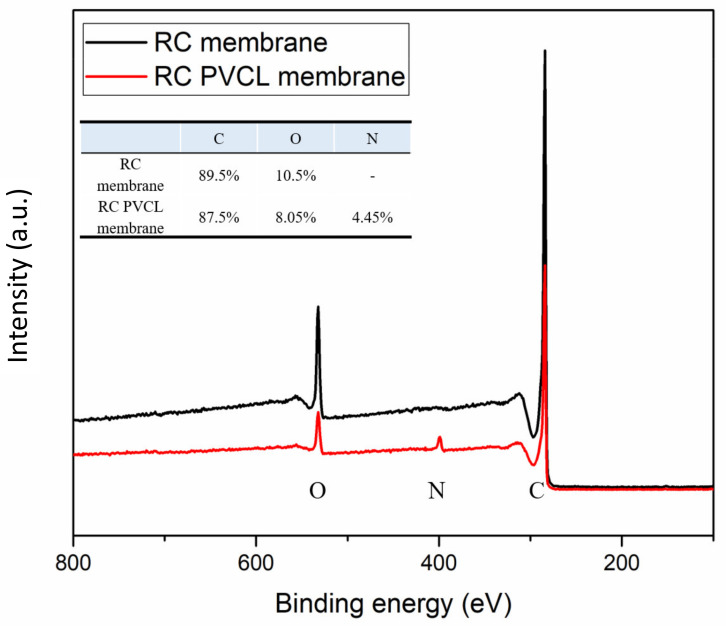
X-ray photoelectron (XPS) spectroscopy of the electrospun RC membranes before and after PVCL ligand grafting.

FTIR, XPS and SEM methods were used to characterize the chemical and structural properties of electrospun membranes before and after surface modification. The FTIR spectra of the originally fabricated cellulose acetate (CA) membrane, the subsequently hydrolyzed RC membrane and PVCL modified membranes are shown in Figure 2. As can be seen, the electrospun CA membrane exhibits a significant characteristic carbonyl (C=O) peak at around 1630 cm^−1^ [8]. As the acetal group was hydrolyzed to the hydroxyl group in the RC membrane, a new broad peak was observed at around 3400 cm^−1^, which is characteristic of the –OH group. In the meantime, the carbonyl peak completely disappeared after hydrolysis indicating the successful completion of the hydrolysis reaction. After grafting the PCVL ligand, a new weaker carbonyl group peak from the ligand reappears, indicating the successful grafting of PVCL on the membrane surface [8]. The stretching vibrations of the N–H and O–H groups of the PVCL chain on the RC membrane correspond to the broad bands at 3000–3700 cm^−1^ [37]. Elemental composition was determined from XPS analysis as shown in Figure 3. The N peak was observed after surface modification with PVCL, indicating successful modification of the ligand.

**Figure 4:**
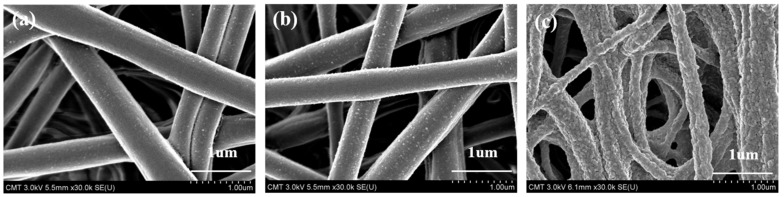
Scanning Electron Microscopy (SEM) images of cellulose acetate (CA) electrospun membrane (**a**), regenerated cellulose (RC) membrane (**b**) and PVCL modified RC membrane (**c**).

Figure 4 shows SEM images of the original electrospun CA membrane as well as the RC membranes after hydrolysis and after surface modification. Based on these SEM images, the average fiber diameter of the membranes was estimated. For the CA membrane, the diameters are estimated to range between 330 and 440 µm, similar to the previously reported results [29]. After hydrolysis converting CA to RC membranes, no significant change in fiber diameter or membrane morphology was observed except that the RC membrane became slightly rougher. However, after surface modification with PVCL, the membrane surface morphology altered significantly with much rougher surfaces. This is partly due to the organic solvent used, which could have affected the fiber morphology, and partly due to the presence of grafted PVCL ligands on the membrane substrate.

**Figure 5:**
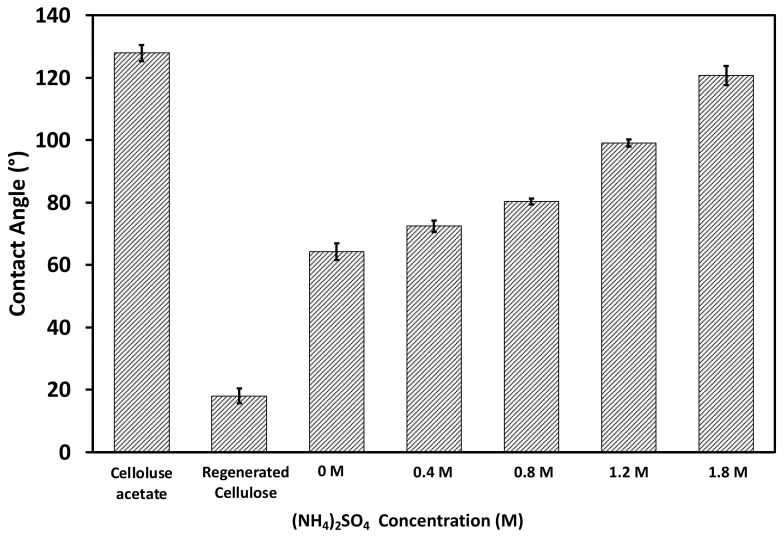
Contact angles of the electrospun CA, the subsequently hydrolyzed RC membrane substrates, as well as PVCL immobilized RC membranes under different concentrations of (NH_4_)_2_SO_4_ salt solutions.

Contact angle measurements using the air bubble method were performed to determine the relative hydrophobicity of the fabricated membranes [38]. The converted water contact angles are shown in Figure 5. The electrospun CA membrane exhibited a contact angle of approximately 130°, whereas the flat-surface CA membrane had a contact angle of nearly 80° [39], which can be attributed to the presence of air gaps in its scaffold-like structure. After hydrolysis and conversion to the RC membrane, the contact angle of the electrospun membrane was reduced considerably to approximately 18°, mainly due to the presence of the hydroxyl groups. After PVCL ligand grafting, the water contact angle was increased to approximately 63°. The PVCL ligand grafted membrane is somewhat more hydrophobic compared to the RC membrane substrate due to the slightly more hydrophobic nature of the PVCL ligand compared to cellulose substrate. Since PVCL is temperature- and salt ion-responsive with its hydrophobicity affected by solution conditions, contact angles were also measured for the functionalized RC membrane by increasing the ionic strength of the ammonium sulfate solution from 0.4 to 1.8 M. The measurements are also shown in Figure 5. It can be seen that the membrane surface becomes more hydrophobic as the salt concentration increases with contact angle increasing from 63° in water to 120° in 1.8 M (NH_4_)_2_SO_4_.

**Figure 6:**
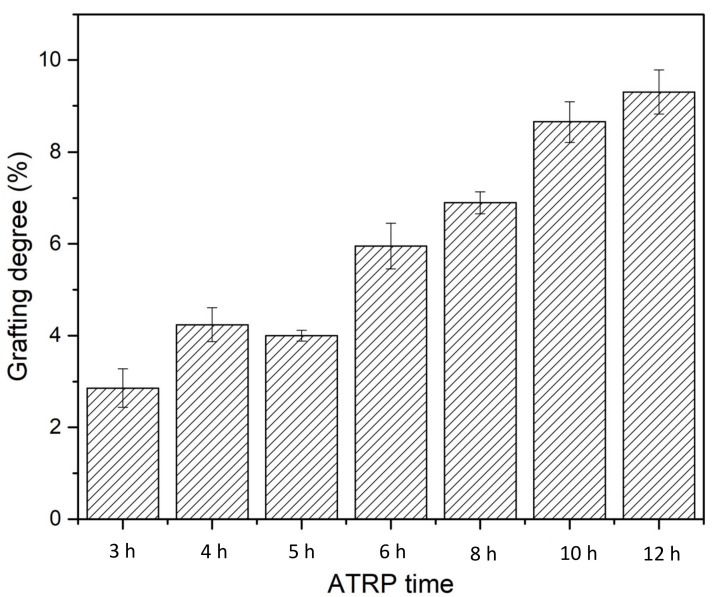
Grafting degree as a function of polymerization time for PVCL immobilized on the electrospun RC membrane substrate.

Grafting degree (GD) was determined based on Equation (1) by calculating the percentage of weight increase compared to the unmodified membrane. As shown in Figure 6, membrane GD increases as the ATRP time increases almost linearly, indicating the well-controlled nature of the polymerization reaction. However, it is still rather challenging to quantify the exact chain length and chain density of the PVCL grafted on the membrane substrate.

### 3.2. Static Binding Capacity

**Figure 7:**
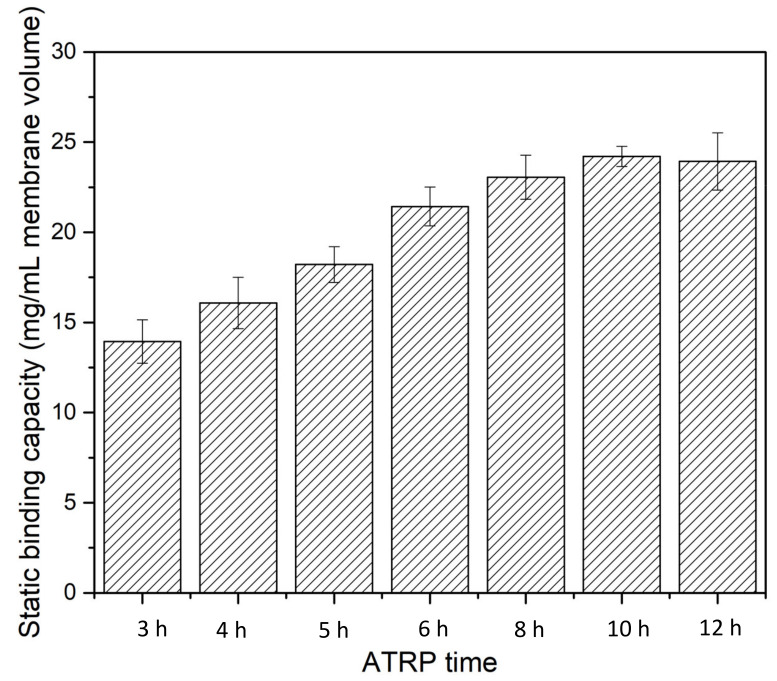
Static binding capacity as a function of polymerization time for PVCL grown on membranes.

Static binding experiments were performed using BSA as the model protein to evaluate the performance of the fabricated HIC membranes. Initially, the static binding capacity of BSA was evaluated as a function of ATRP grafting time for 1 g/L BSA in 20 mM phosphate buffer with 1.8 M ammonium sulfate, as shown in Figure 7. The concentrations of BSA were measured after 5 h of equilibrium. The results show that static binding capacity increases as ATRP time increases, indicating that longer polymer chains have higher binding capacity. However, after more than 6 h of polymerization, further enhancement of binding capacity is not significant, increasing from ~22 mg/mL MV at 6 h to 25 mg/mL MV at 10 h. Further increases in ATRP time led to a slight decrease in BSA binding capacity. For the subsequent experiments, an ATRP time of 6 h was used for both static and dynamic binding studies.

**Figure 8:**
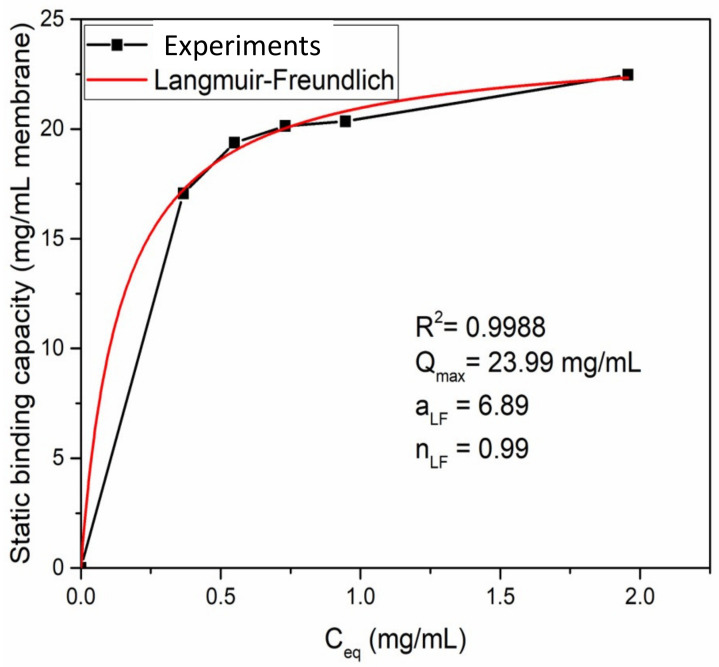
Langmuir–Freundlich curve for ATRP of a 6 h PVCL modified membrane.

The adsorption isotherm was investigated for 1 g/L BSA in 1.8 M ammonium sulfate buffer solution as shown in Figure 8. A Langmuir–Freundlich isotherm model [40,41] was able to describe the adsorption behavior using the following equation:

(4) $$q=\frac{Q_{\max}{\left(a_{\mathrm{LF}}C_{\mathrm{eq}}\right)}^{n_{\mathrm{LF}}}}{1+{\left(a_{\mathrm{LF}}C_{\mathrm{eq}}\right)}^{n_{\mathrm{LF}}}}$$

where q represents the amount of proteins adsorbed at equilibrium (mg/mL MV), Q_max_ represents the maximum adsorption capacity of a membrane (mg/mL MV), C_eq_ represents the protein concentration at equilibrium (mg/mL), a_LF_ represents the adsorption affinity constant (mL/mg) and n_LF_ represents the heterogeneity index. In this study, a fitting R^2^ value of 0.9988 was obtained indicating a high-quality fit. The fitting parameters are shown in Figure 8. It can be seen that Q_max_ is about 24 mg/mL MV which is in agreement with earlier results. The heterogeneity index n_LF_ is 0.99, indicating that BSA adsorption by the electrospun HIC membrane follows a Langmuir isotherm model with monolayer adsorption.

### 3.3. Protein Dynamic Binding Capacity and Recovery

Experiments were conducted to evaluate the performance of the fabricated HIC membranes under actual chromatographic runs. The experiments were run with a range of BSA feed concentrations ranging from 0. 1 to 1 g/L BSA to determine protein dynamic binding capacity (DBC) and recovery. Initially, the electrospun RC membranes grafted with PVCL at two different chain lengths were tested for BSA binding and recovery, as shown in Table 1. At GD 6% (6 h ATRP) and 9% (10 h ATRP), DBC and recovery are quite different at the same BSA concentration. At 6% GD, a DBC of 8.0 mg/mL MV and a recovery of 98.2% can be achieved when the BSA concentration is 0.1 g/L. As the BSA concentration increased to 1.0 g/L, the DBC reached 30.7 mg/mL MV. However, recovery reduced to only 51.5%. At 9% GD, a DBC of 14.3 and 43.4 mg/mL MV was obtained at 0.1 and 1.0 g/L BSA feed solution, respectively, much higher than the corresponding values at 6% GD. However, recovery was only at 80.6% and 21.1%, respectively, significantly reduced compared to the values at 6% GD. Again, we can see that the longer polymer chain tends to have a higher DBC but also leads to reduced product recovery consistent with previous results [8]. Longer polymer chains lead to increased coverage of the ligand on the membrane surface and inside the membrane pores for protein binding which in turn leads to higher binding capacity. However, longer polymer chains with higher surface/pore coverage of the grafted polymer could also trap the proteins and prevent them from eluting, thereby leading to lower recovery. For subsequent studies, membranes with 6 h ATRP time were investigated.

In addition to the effect of polymer chain length on protein binding and elution, the effect of ligand density on DBC and product recovery was also investigated. Chromatographic runs for electrospun membranes with different ligand densities were performed. The variation of chain densities was achieved by immobilizing BIB initiator for 3 h at 40, 80 and 200 mM. Two sets of experiments were performed for each condition, with the final result as an average of the two. As shown in Table 2, the functionalized electrospun membrane using 80 mM initiator has the highest DBC of 8.0 and 30.7 mg/mL MV at 0.1 and 1.0 g/L BSA loading conditions, respectively. With 40 mM initiator and lower ligand density, DBCs are 4.8 and 25.0 mg/mL MV for the two protein feed conditions, respectively. Increasing initiator concentration to 200 mM and with higher ligand density, the DBC values are slightly reduced to 6.2 and 28.4 mg/mL MV, respectively. With regard to product recovery, at 40 and 80 mM initiator concentrations with relatively lower ligand densities, recovery at around 98% was achieved at 0.1 g/L BSA feed concentration. When feed concentration increases to 1.0 g/L, recovery is reduced to 57.2% and 51.5% for the two ligand densities. At the highest ligand density with 200 mM initiator concentration, the recovery is only 83.4% for 0.1 g/L BSA feed and 41.4% for 1 g/L feed. It can be seen that protein dynamic binding capacity of functionalized electrospun HIC membranes is significantly higher than the DBC of the functionalized flat sheet HIC membrane at similar conditions [5,6,7,8,10,23]. Previous studies [8] achieved an optimal dynamic binding capacity of 12.6 mg/mL MV and a recovery of 78% with 1.0 g/L BSA feed on HIC ligand functionalized on the flat sheet membrane. A binding capacity of 30.7 mg/mL MV can be achieved on electrospun membranes with a slightly lower recovery of 51.5% at the same feed condition. It seems that there is a trade-off between binding capacity and recovery [8]. High binding capacity leads to lower recovery which is in agreement with previous observations. Of the three ligand densities, 80 mM initiator concentration appears to provide the best compromise between binding capacity and recovery. Therefore, the ligand density with 80 mM initiator concentration was used.

The effect of the amount of protein loaded on the functionalized membrane on DBC and recovery was also investigated systematically. BSA feed streams in 20 mM phosphate buffer with 1.8 M (NH4)_2_SO_4_ at 0.1, 0.3, 0.5 and 1.0 g/L concentrations were loaded on the functionalized HIC membranes. These membranes had a GD of 6% functionalized by a 3 h immobilization in 80 mM initiator followed by a 6 h ATRP reaction. Table 3 shows the DBC and recovery at different BSA loading concentrations. It can be seen that as BSA concentration increases DBC increases, whereas recovery decreases. This agrees with previous observations that higher binding capacity typically correlates with lower protein recovery for a given membrane. Nevertheless, a moderate DBC of 16.6 mg/mL MV and a recovery of 75.7% can be achieved when BSA loading concentration is 0.5 g/L.

The effect of flow rate on dynamic protein binding capacity and recovery is shown in Table 4. In previous studies, flow rate was kept at 1.0 mL/min. Here, flow rates of 0.5 and 2 mL/min were investigated. It can be seen from Table 4 that as flow rate decreases from 1 to 0.5 mL/min, DBC decreases slightly from 30.7 to 28.9 mg/mL MV, whereas recovery increases from 51.5% to 57.5%. When flow rate increases from 1 to 2 mL/min, both DBC and recovery decrease to 27.4 mg/mL MV and 41.7%, respectively. It is clear that flow rate has a strong effect on product recovery since the elution and migration of the protein from the binding sites are affected by both hydrodynamic and diffusive forces. The slow flow rate will allow the protein molecules to migrate out of the membrane. On the other hand, the effect of flow rate on DBC only slightly indicates that it is possible to improve the performance of these functionalized responsive HIC membranes by reducing flow rate.

The effect of electrospun fiber diameter on protein binding capacity and recovery was also investigated, as shown in Table 5. The concentration of the polymer solution for electrospinning was kept the same at 13.8%. However, the voltage applied during electrospinning increased from 12.5 to 20 kV, which decreased fiber diameter from 330–440 to 220–350 μm as shown in Table 5. The pore size of the membrane formed also decreased from 3.24 to 2.88 μm. The modification conditions for these two membranes remained at 80 mM initiator concentration and 6 h ATRP time. The membrane with smaller fibers exhibited a smaller average pore size. The DBCs are 8.0 and 9.8 mg/mL MV for membranes with larger and smaller fiber diameters, respectively, when the BSA loading concentration is 0.10 g/L. Recovery was 98% for the larger fiber and was reduced to 79% for the smaller fiber. It seems that the small gain in binding capacity is compensated by the significant reduction in recovery. At a high BSA loading concentration of 1.0 g/L, the DBCs are 30.7 and 33.6 mg/mL MV, respectively, for the larger and smaller fiber membranes. However, recovery is reduced from 51.5% to only 37.9%. Clearly, larger fiber diameter membranes have better overall performance for bind-and-elute applications of these responsive HIC membranes.

Finally, binding capacity and recovery of IgG_4_ were investigated using our responsive HIC membranes at three different loading concentrations of 0.1, 0.5 and 1 g/L. The membrane (80 mM initiator, 6 h ATRP) and feed buffer (20 mM phosphate buffer with 1.8 M (NH_4_)_2_SO_4_) conditions remained the same as the previously performed experiments. The results are shown in Table 6. Average binding capacities of 16.3, 28.8 and 47.8 mg/mL MV were obtained at 0.1, 0.5 and 1.0 g/L IgG loading concentrations. However, recovery decreased from 85.3% to 24.0% and finally to 14.2% as IgG concentration increased. It is clear that IgG has an overall higher binding capacity compared to BSA. However, its recovery is much reduced compared to that of BSA, particularly at high protein concentrations. Himstedt et al. [23] observed an IgG capacity of 21 mg/mL, whereas Liu et al. [6] reported an IgG capacity of 12 mg/mL using flat sheet PVCL functionalized membranes with 1 g/L IgG loading concentration. These results highlight the potential benefits derived from using an open and more porous electrospun membrane as a substrate for ligand functionalization.

## 4. Conclusions

Responsive HIC membranes were successfully fabricated by functionalizing electrospun RC membrane substrate. The PVCL functionalized electrospun membranes exhibit a much higher binding capacity compared to the corresponding flat sheet membranes. A maximum static BSA binding capacity of ~25 mg/mL MV can be achieved. The longer polymer chains lead to higher static binding capacity up to a grafting degree of 6%. Binding capacity also increases with salt concentration which is in agreement with previous studies. Static binding follows the monolayer Langmuir adsorption isotherm.

For the dynamic binding studies, protein binding capacity increased with protein concentration while protein recovery decreased as its concentration increased. There is a clear trade-off between binding capacity and recovery rate. There is an optimal chain density for the performance of these responsive HIC membranes. In addition, it also appears that larger fiber diameter leads to slightly reduced protein binding capacity but significantly higher recovery, indicating better performance for electrospun membranes with thicker fibers. This is due to the larger pores that resulted from the thicker fibers. There is some influence of flow rate on protein binding and elution. A slow flowrate (e.g., 0.5 mL/min) leads to slightly reduced binding capacity but slightly enhanced recovery compared to the corresponding values at 1 mL/min. On the other hand, a higher flowrate (e.g., 2 mL/min) leads to both slightly reduced binding capacity and much reduced recovery. The performance of these responsive HIC membranes is protein dependent due to their different hydrophobicity and other properties. The IgG_4_ tested showed much higher binding capacity compared to BSA at the same conditions but highly reduced protein recovery.

## Figures and Tables

**Table 1:** The effect of grafting degree on dynamic binding capacity (DBC) and recovery of bovine serum albumin (BSA) at two protein loading concentrations.

Grafting Degree (DG) (%)	BSA Loading Concentration (g/L)	Protein Binding Capacity (mg/mL)	Recovery (%)
6	0.10	7.95 ± 0.75	98.17 ± 0.02
6	1.0	30.69 ± 0.34	51.54 ± 1.96
9	0.10	14.29	80.64
9	1.0	43.43	21.09

**Table 2:** The effect of ligand density and BSA concentration on DBC and recovery.

Initiator (mM)	BSA Loading Concentration (g/L)	Protein Binding Capacity (mg/mL)	Recovery (%)
40	0.1	4.79 ± 0.11	97.54 ± 2.27
40	1.0	24.95 ± 0.56	57.24 ± 2.33
80	0.1	7.95 ± 0.75	98.17 ± 0.02
80	1.0	30.69 ± 0.34	51.54 ± 1.96
200	0.1	6.22 ± 0.11	83.39 ± 0.22
200	1.0	28.38 ± 1.75	41.44 ± 0.01

**Table 3:** The effect of protein feed concentration on DBC and product recovery for PVCL grafted with 80 mM initiator and 6 h ATRP.

BSA Loading Concentration (g/L)	Protein Binding Capacity (mg/mL)	Recovery (%)
0.1	7.95 ± 0.75	98.17 ± 0.02
0.3	9.93	91.56
0.5	16.55 ± 2.09	75.67 ± 3.32
1.0	30.69 ± 0.34	51.54 ± 1.96

**Table 4:** The effect of flow rate on DBC and recovery of BSA for PVCL grafted with 80 mM initiator and 6 h ATRP.

BSA Loading Concentration (g/L)	Flowrate (mL/min)	Protein Binding Capacity (mg/mL)	Recovery (%)
1	0.5	28.87 ± 0.79	57.51 ± 1.23
1	1	30.69 ± 0.34	51.54 ± 1.96
1	2	27.36 ± 0.93	41.72 ± 1.80

**Table 5:** The effect of fiber diameter on DBC and recovery.

Voltage (kV)	Fiber Diameter (nm)	Pore Size (μm)	BSA (g/L)	DBC (mg/mL)	Recovery (%)
12.5	330–440	3.24 ± 0.27	0.1	7.95 ± 0.75	98.17 ± 0.02
			1.0	30.69 ± 0.34	51.54 ± 1.96
20	220–350	2.88 ± 0.39	0.1	9.84	79.08
			1.0	33.58	37.86

**Table 6:** Dynamic protein binding capacity and recovery results for IgG_4_.

IgG_4_ Loading Concentration (g/L)	Protein Binding Capacity (mg/mL)	Recovery (%)
0.1	16.31 ± 0.23	85.32 ± 1.38
0.5	28.78 ± 0.18	23.95 ± 1.17
1	47.68 ± 1.29	14.22 ± 1.93

## Data Availability

The data presented in the study are already shown in the tables from this article.

## References

[1] Cramer S.M., Holstein M.A. (2011). Downstream bioprocessing: Recent advances and future promise. Curr. Opin. Chem. Eng..

[2] Liu Z., Wickramasinghe S.R., Qian X. (2017). Membrane chromatography for protein purifications from ligand design to functionalization. Sep. Sci. Technol..

[3] Devi N., Patel S.K.S., Kumar P., Singh A., Thakur N., Lata J., Pandey D., Thakur V., Chand D. (2022). Bioprocess Scale-up for Acetohydroxamic Acid Production by Hyperactive Acyltransferase of Immobilized Rhodococcus Pyridinivorans. Catal. Lett..

[4] Eriksson K.O. (2018). Hydrophobic Interaction Chromatography, Biopharmaceutical Processing.

[5] Ghosh R. (2001). Separation of proteins using hydrophobic interaction membrane chromatography. J. Chromatogr. A.

[6] Liu Z., Wickramasinghe S.R., Qian X. (2017). Ion-specificity in protein binding and recovery for the responsive hydrophobic poly (vinylcaprolactam) ligand. RSC Adv..

[7] Lienqueo M.E., Mahn A., Salgado J.C., Asenjo J.A. (2007). Current insights on protein behaviour in hydrophobic interaction chromatography. J. Chromatogr. B.

[8] Liu Z., Wickramasinghe S.R., Qian X. (2017). The architecture of responsive polymeric ligands on protein binding and recovery. RSC Adv..

[9] Baumann P., Baumgartner K., Hubbuch J. (2015). Influence of binding pH and protein solubility on the dynamic binding capacity in hydrophobic interaction chromatography. J. Chromatogr. A.

[10] Vu A., Qian X., Wickramasinghe S.R. (2017). Membrane-based hydrophobic interaction chromatography. Sep. Sci. Technol..

[11] Liu Z., Du H., Wickramasinghe S.R., Qian X. (2014). Membrane surface engineering for protein separations: Experiments and simulations. Langmuir.

[12] Boi C., Malavasi A., Carbonell R.G., Gilleskie G. (2020). A direct comparison between membrane adsorber and packed column chromatography performance. J. Chromatogr. A.

[13] Chenette H.C., Robinson J.R., Hobley E., Husson S.M. (2012). Development of high-productivity, strong cation-exchange adsorbers for protein capture by graft polymerization from membranes with different pore sizes. J. Membr. Sci..

[14] Chen S.-T., Wickramasinghe S.R., Qian X. (2020). Electrospun weak anion-exchange fibrous membranes for protein purification. Membranes.

[15] Hall T., Kelly G.M., Emery W.R. (2018). Use of mobile phase additives for the elution of bispecific and monoclonal antibodies from phenyl based hydrophobic interaction chromatography resins. J. Chromatogr. B.

[16] Sun X., Qian X. (2019). Atomistic Molecular Dynamics Simulations of the Lower Critical Solution Temperature Transition of Poly (N-vinylcaprolactam) in Aqueous Solutions. J. Phys. Chem. B.

[17] Zhang Y., Furyk S., Bergbreiter D.E., Cremer P.S. (2005). Specific ion effects on the water solubility of macromolecules: PNIPAM and the Hofmeister series. J. Am. Chem. Soc..

[18] Du H., Wickramasinghe R., Qian X. (2010). Effects of salt on the lower critical solution temperature of poly (N-isopropylacrylamide). J. Phys. Chem. B.

[19] Du H., Qian X. (2012). The Interactions between Salt Ions and Thermo-Responsive Poly (N-Isopropylacrylamide) from Molecular Dynamics Simulations. Responsive Membr. Mater..

[20] Du H., Wickramasinghe S.R., Qian X. (2013). Specificity in cationic interaction with poly (N-isopropylacrylamide). J. Phys. Chem. B.

[21] Zhang Y., Furyk S., Sagle L.B., Cho Y., Bergbreiter D.E., Cremer P.S. (2007). Effects of Hofmeister anions on the LCST of PNIPAM as a function of molecular weight. J. Phys. Chem. C.

[22] Hiruta Y., Nagumo Y., Suzuki Y., Funatsu T., Ishikawa Y., Kanazawa H. (2015). The effects of anionic electrolytes and human serum albumin on the LCST of poly (N-isopropylacrylamide)-based temperature-responsive copolymers. Colloids Surf. B Biointerfaces.

[23] Himstedt H.H., Qian X., Weaver J.R., Wickramasinghe S.R. (2013). Responsive membranes for hydrophobic interaction chromatography. J. Membr. Sci..

[24] Darvishmanesh S., Qian X., Wickramasinghe S.R. (2015). Responsive membranes for advanced separations. Curr. Opin. Chem. Eng..

[25] Okubo K., Ikeda K., Oaku A., Hiruta Y., Nagase K., Kanazawa H. (2018). Protein purification using solid-phase extraction on temperature-responsive hydrogel-modified silica beads. J. Chromatogr. A.

[26] Nagase K., Ishii S., Ikeda K., Yamada S., Ichikawa D., Akimoto A.M., Hattori Y., Kanazawa H. (2020). Antibody drug separation using thermoresponsive anionic polymer brush modified beads with optimised electrostatic and hydrophobic interactions. Sci. Rep..

[27] Nomoto D., Nagase K., Nakamura Y., Kanazawa H., Citterio D., Hiruta Y. (2021). Anion species-triggered antibody separation system utilizing a thermo-responsive polymer column under optimized constant temperature. Colloids Surf. B Biointerfaces.

[28] Huang L., Arena J.T., McCutcheon J.R. (2016). Surface modified PVDF nanofiber supported thin film composite membranes for forward osmosis. J. Membr. Sci..

[29] Chitpong N., Husson S.M. (2017). Polyacid functionalized cellulose nanofiber membranes for removal of heavy metals from impaired waters. J. Membr. Sci..

[30] Fu Q., Wang X., Si Y., Liu L., Yu J., Ding B. (2016). Scalable fabrication of electrospun nanofibrous membranes functionalized with citric acid for high-performance protein adsorption. ACS Appl. Mater. Interfaces.

[31] Dods S.R., Hardick O., Stevens B., Bracewell D.G. (2015). Fabricating electrospun cellulose nanofibre adsorbents for ion-exchange chromatography. J. Chromatogr. A.

[32] Zhang H., Wu C., Zhang Y., White C.J.B., Xue Y., Nie H., Zhu L. (2010). Elaboration, characterization and study of a novel affinity membrane made from electrospun hybrid chitosan/nylon-6 nanofibers for papain purification. J. Mater. Sci..

[33] Ma H., Hsiao B.S., Chu B. (2013). Electrospun nanofibrous membrane for heavy metal ion adsorption. Curr. Org. Chem..

[34] Ma Z., Lan Z., Matsuura T., Ramakrishna S. (2009). Electrospun polyethersulfone affinity membrane: Membrane preparation and performance evaluation. J. Chromatogr. B.

[35] Schneiderman S., Zhang L., Fong H., Menkhaus T.J. (2011). Surface-functionalized electrospun carbon nanofiber mats as an innovative type of protein adsorption/purification medium with high capacity and high throughput. J. Chromatogr. A.

[36] Chen S.-T., Wickramasinghe S.R., Qian X. (2021). High Performance Mixed-Matrix Electrospun Membranes for Ammonium Removal from Wastewaters. Membranes.

[37] Li G., Xiao J., Zhang W. (2011). Knoevenagel condensation catalyzed by a tertiary-amine functionalized polyacrylonitrile fiber. Green Chem..

[38] Chiao Y.-H., Sengupta A., Chen S.-T., Huang S.-H., Hu C.-C., Hung W.-S., Chang Y., Qian X., Wickramasinghe S.R., Lee K.-R. (2019). Zwitterion augmented polyamide membrane for improved forward osmosis performance with significant antifouling characteristics. Sep. Purif. Technol..

[39] Du Y., Li Y., Wu T. (2017). A superhydrophilic and underwater superoleophobic chitosan–TiO_2_ composite membrane for fast oil-in-water emulsion separation. RSC Adv..

[40] Umpleby R.J., Baxter S.C., Chen Y., Shah R.N., Shimizu K.D. (2001). Characterization of molecularly imprinted polymers with the Langmuir–Freundlich isotherm. Anal. Chem..

[41] Jeppu G.P., Clement T.P. (2012). A modified Langmuir-Freundlich isotherm model for simulating pH-dependent adsorption effects. J. Contam. Hydrol..

